# Establishment of a Spontaneous Liver Fibrosis Model in NOD/SCID Mice Induced by Natural Aging

**DOI:** 10.3390/biology12121493

**Published:** 2023-12-06

**Authors:** Lihua Qiu, Zhaoxia Ma, Jianxiu Sun, Zhen Wu, Mengting Wang, Sitao Wang, Yunhui Zhao, Shu Liang, Min Hu, Yanjiao Li

**Affiliations:** Yunnan Key Laboratory for Basic Research on Bone and Joint Diseases & Yunnan Stem Cell Translational Research Center, Kunming University, Kunming 650214, China; lihuaqiu666@126.com (L.Q.); zxma@kmu.edu.cn (Z.M.); ts15288530428@163.com (J.S.); wuzhenup@163.com (Z.W.); wmt1920228750@163.com (M.W.); 18287353315@163.com (S.W.); 18314305871@163.com (Y.Z.); 18288215906@163.com (S.L.)

**Keywords:** liver fibrosis, aging, spontaneous model, NOD/SCID mice

## Abstract

**Simple Summary:**

Liver fibrosis is a chronic disease that can lead to serious health problems, including liver cancer. However, the pathogenesis of liver fibrosis caused by natural aging is not fully understood. For researchers studying liver fibrosis, an important tool for conducting mechanism research and testing anti-liver fibrosis therapies is an in vivo model that can reflect the pathological causes of liver fibrosis. This study aimed to establish a model of spontaneous liver fibrosis in NOD/SCID mice induced by natural aging. This model’s validity was confirmed using cutting-edge fibrosis-related measurements. This study provides a useful tool for further research into the causes and potential treatments for liver fibrosis. The findings may help to develop new therapies for chronic liver diseases which affect millions of people worldwide.

**Abstract:**

Liver fibrosis, a critical pathological feature of chronic liver diseases, arises from a multitude of pathogenic factors. Consequently, establishing an appropriate animal model to simulate liver fibrosis holds immense significance for comprehending its underlying pathogenesis. Despite the numerous methodologies available for generating liver fibrosis models, they often deviate substantially from the spontaneous age-related liver fibrosis process. In this study, compared with young (12 weeks) and middle-aged NOD/SCID mice (32 weeks), there were a large number of fibrous septum and collagen in the liver tissue of old NOD/SCID mice (43 weeks, 43 W). Immunohistochemical analysis unequivocally indicated heightened α-SMA content within the liver tissue of the 43 W mice, thereby underscoring aging’s role in triggering the epithelial-to-mesenchymal transition. In addition, SA-β-gal staining as well as P21 expression were increased, and SIRT1 and SIRT3 expression were decreased in 43 W mice. A comprehensive evaluation encompassing transmission electron microscopy and fluorescence quantitative analysis elucidated compromised mitochondrial function and reduced antioxidant capacity in hepatocytes of the 43 W mice. Furthermore, the aging process activated the pro-fibrotic TGF-β-SMAD pathway, concurrently inducing hepatocellular inflammation. The results of the present study not only validate the successful construction of a spontaneous liver fibrosis mouse model through natural aging induction but also provide initial insights into the mechanisms underpinning age-induced liver fibrosis.

## 1. Introduction

Over the past century, human life expectancy has witnessed a remarkable increase, triggering a significant surge in the global elderly population. Projections indicate that the number of individuals aged 65 and above will surge from 524 million in 2010 to nearly 1.5 billion by 2050 [1]. As individuals age, their capacity to maintain homeostasis gradually diminishes, rendering them more vulnerable to external stressors or injuries [2]. Extensive prior research has illuminated the connection between aging and progressive, wide-ranging impairment of cellular functions, representing a key risk factor for various chronic ailments such as inflammatory diseases, diabetes, and cancer [3]. Furthermore, aging exposes individuals to structural and functional harm to the liver, accompanied by assorted transformations in hepatocytes, culminating in the emergence of age-related liver disorders [4,5]. Additionally, aging heightens susceptibility to fibrotic responses, culminating in liver fibrosis as a recurrent end stage across most chronic liver conditions [6,7].

Liver fibrosis emerges as an outcome of an excessive healing response stemming from prolonged liver injury, encompassing a remarkably intricate and progressive process chiefly discernible in the disrupted intracellular substance equilibrium provoked by persistent hepatic inflammation [8,9]. The process involves recurrent destruction and regeneration of hepatocytes, culminating in the immoderate accumulation and anomalous distribution of extracellular matrix (ECM) constituents such as collagen and fibronectin within the liver [10]. ECM is produced by myofibroblasts, which are transformed from epithelial cells or resident interstitial fibroblasts, a process called epithelial-to-mesenchymal transition (EMT) [11]. In the wake of fibrosis, the liver’s normal architecture is disrupted, hepatocytes are lost, and the liver’s ordinary synthetic and metabolic functions are compromised. Liver fibrosis constitutes a crucial hallmark of chronic liver disease, serving as a pivotal stage in the progression from chronic liver disease to cirrhosis. Cirrhosis is closely associated with elevated morbidity and mortality rates and significantly contributes to primary liver cancer [12]. While liver fibrosis evolves gradually and is potentially reversible at a histological level, advanced liver cirrhosis is notably challenging to revert. Consequently, the timely interception of chronic liver fibrosis stands as a pivotal strategy in the prevention and treatment of chronic liver diseases [13].

For researchers delving into liver fibrosis, an essential tool for mechanistic studies and the testing of anti-fibrotic therapies lies in an in vivo model that mirrors the pathogenesis of liver fibrosis. This model also serves as a basis for clinical translation. In this pursuit, factors such as cost, practicality, and the availability of comprehensive genetic information play a pivotal role. Accordingly, the preferred choice often leans towards small rodents, as highlighted by Weiler-Normann et al. [7]. In the study of autologous stem cells in the treatment of liver disease, in addition to the liver disease models replicated by wild-type C57BI6 mice, it is also necessary to study the feasibility of immunodeficient animals as liver disease models, so as to study the intervention of human stem cells in immunodeficient animal liver disease models on the premise of avoiding immune rejection. The modeling methods for liver fibrosis include hepatotoxicity induction, high-fat diet induction, immunization, bile duct obstruction, the parasitic method, and the transgenic method [10]. While these methods offer diverse pathways to create fibrotic models, a successful model must faithfully mirror the characteristics and pathogenesis of the human disease. The human liver fibrosis caused by aging has progressed over decades; collagen is gradually cross-linked, rendering it resistant to dissolution and distorting the liver’s architecture. It is worth noting that replicating these key features within animal models established through the aforementioned methods might present challenges. However, a parallel can be drawn between liver fibrosis in aging NOD/SCID mice and in aging humans—both primarily attributed to age-related factors. This consistency aligns with the fundamental etiology in the pattern of disease onset and progression. Addressing these considerations, this study undertook the establishment of a spontaneous liver fibrosis model in NOD/SCID mice induced by the natural aging process. This model’s validity was confirmed using cutting-edge fibrosis-related measurements. The overarching aim was to furnish theoretical significance and a practical reference for the clinical management of age-related liver fibrosis in humans.

## 2. Materials and Methods

### 2.1. Animal Preparation

NOD/SCID male mice were sourced from Jiangsu GemPharmatech LLC (Nanjing, China). The mice were individually housed in cages, maintaining a 12-h light/dark cycle and providing them unrestricted access to both food and water. The composition of feed was 1 kg feed: ≤100 g of moisture, ≥180 g of crude protein, ≥40 g of crude fat, ≤50 g of crude fiber, ≤80 g of crude ash, 10–18 g of calcium, 6–12 g of total phosphorus, ≥8.2 g of lysine, and ≥5.3 g of methionine + cystine. They were kept in controlled environments, free from specific pathogens, and maintained at a controlled temperature (approximately 25 °C) and relative humidity (40–60%). The mice were humanely euthanized upon reaching 12 weeks of age (12 W, young mice, *n* = 6, 25.28 ± 1.44 g), 32 weeks of age (32 W, middle-aged mice, *n* = 6, 28.53 ± 1.75 g), and 43 weeks of age (43 W, senior mice, *n* = 6, 23.80 ± 1.12 g). The 43 W mice constituted the model group, while the 12 W and 32 W mice comprised the control groups. Before being euthanized, mice were fasted overnight. We used carbon dioxide (CO_2_) to euthanize all mice. CO_2_-mediated euthanasia provides a rapid, painless, stress-free death because CO_2_ overdose causes rapid unconsciousness followed by death. To perform euthanasia, the mice that needed to be sacrificed were placed into the euthanasia chamber, where the CO_2_ flow rate displaced 30% of the chamber volume per minute. The gas flow was maintained for at least 1 min after apparent clinical death. Liver tissues were harvested for subsequent analysis.

### 2.2. Liver Morphology

The samples of liver tissue were fixed in 4% paraformaldehyde and subjected to a series of alcohol-based dehydration steps. Following this, they were embedded in paraffin and cut into thin slices (3 μm in thickness). These sections underwent a deparaffinization and rehydration process to facilitate H&E (hematoxylin and eosin) staining. Images were captured using the automated slide scanning system (VS200, Olympus, Japan). Pathological grading was performed following the guidelines outlined in the Xi’an Conference (2000) of the Chinese Society of Hepatology, Chinese Medical Association [14]. For interstitial fibrosis, Masson’s trichrome staining was conducted to visualize collagen deposition within the liver tissue. Quantification of fibrotic areas involved calculating the ratio of collagen deposition (blue color area) to the overall field area across three randomly selected regions on each slide using Image J software (version 1.52a, National Institutes of Health, Bethesda, MD, USA).

### 2.3. Immunohistochemistry (IHC) Staining

Formal-fixed, paraffin-embedded liver tissues were sliced into sections with a thickness of 5 μm, then dewaxed in xylene, and dehydrated in alcohol. Subsequently, these sections were blocked by 2% bovine serum albumin followed by incubation with a primary antibody targeting alpha smooth muscle actin (anti-α-SMA; 1:100; ab5694, Abcam, Cambridge, MA, USA) in a humid chamber overnight at 4 °C. Afterward, the sections were thoroughly washed with PBS and incubated with a biotinylated secondary antibody for approximately 30 min at room temperature. The 3,3′-diaminobenzidine tetrahydrochloride (DAB) was applied to visualize the α-SMA expression and the sections were counterstained with hematoxylin. The α-SMA positive areas within the fibrotic region were captured using a slide scanner. The results were calculated from five fields for each liver slice using Image J software.

### 2.4. Senescence-Associated β-Galactosidase (SA-β-gal) Staining

Liver tissues from mice were rapidly frozen and embedded in optimal cutting temperature compound (Sakura Finetek USA, Torrance, CA, USA). These tissue blocks were sectioned using a cryostat microtome (ThermoFischer Scientific, Waltham, MA, USA), resulting in thin slices with a thickness of 10 μm. Following this, the obtained cryosections were affixed onto glass slides. SA-β-gal staining was performed using a senescence detection kit (Beyotime, Shanghai, China) following the stipulated protocols by the manufacturer. The presence of SA-β-gal-positive cells was assessed using a slide scanner.

### 2.5. Transmission Electron Microscopy (TEM)

To perform the TEM, the liver tissues were cut into small 1 mm^3^ pieces and fixed in 2.5% glutaraldehyde buffered in 0.1 mol/L phosphate buffer at pH 7.2 for overnight at 4 °C. The samples were further immersed in 1% osmium tetroxide for a 2 h duration, also at 4 °C. Subsequent steps involved the dehydration process using a graded ethanol series, followed by embedding in the SPI-Pon 812 resin. The embedded samples underwent polymerization at 60 °C for 48 h. For subsequent analyses, semithin sections measuring 800 nm and ultrathin sections measuring 60 nm were obtained using a Leica EM UC7 ultracut microtome (Wetzlar, Germany). Ultrathin sections were dropped onto a Cu grid for 5 min and double stained with 2% uranyl acetate followed by lead citrate and finally imaged on a JEM-1400 Plus transmission electron microscope operated at 80 kV (Japan Electron Optics Laboratory Co., Ltd., Tokyo, Japan).

### 2.6. Hepatic Hydroxyproline Assessment

Measurement of hydroxyproline content was performed using the hydroxyproline detection kit (Nanjing Jiancheng Bioengineering Institute, Nanjing, China) according to the manufacturer’s instructions. In brief, approximately 200 mg of liver specimens were hydrolyzed at 120 °C for 2 h, followed by subsequent lyophilization. The absorption was measured in triplicate at 550 nm using a Multiskan Spectrum (Tecan Austria GmbH, Grodig, Austria). Quantification of hydroxyproline content was carried out by utilizing a standard curve, and the results were expressed as μg hydroxyproline/mg liver.

### 2.7. RNA Extraction and Real-Time PCR

Total RNA was extracted from liver tissues of mice of varying ages using the TRIzol (Invitrogen, Carlsbad, CA, USA). The extracted RNA was then subjected to reverse transcription utilizing the RT reagent kit (Takara, Dalian, China) for mRNA expression analysis. We scrutinized the expression fluctuations of pivotal coding genes associated with aging-related factors (P21, P53), anti-fibrosis factors (SIRT1, SIRT3), pro-inflammatory factors (IL1β, IL6, IL8, TNF-α), pro-fibrosis factors (TGF-β, SMAD3), and antioxidant factors (SOD1, SOD2). Primer sequences were designed by Primer Premier 5.0 [15] and are outlined in Table S1. The qPCR was carried out by QuantStudio Q5 System (Applied Biosystems, Waltham, MA, USA). The Dib^®^ SYBR qPCR SuperMix Plus (Hong Kong Aibisheng Biotechnology Co., Ltd., Hong Kong, China) functioned as the fluorescent dye for the qPCR reaction. In order to ensure the purity of the PCR product, melting curve analysis was performed. Endogenous control ACTB was employed to normalize the expression levels of individual target genes. The qPCR data were analyzed through the 2^−ΔΔCt^ method.

### 2.8. Statistical Analyses

Experimental results were presented as mean ± SEM for each group. Statistical analysis and graphical representation were executed using GraphPad Prism 5 software (GraphPad Software Inc., La Jolla, CA, USA). The statistical significance of differences between groups was ascertained through one-way ANOVA, with *p* values less than 0.05 considered significant.

## 3. Results

### 3.1. Hepatic Pathology in Mouse Liver

**Figure 1:**
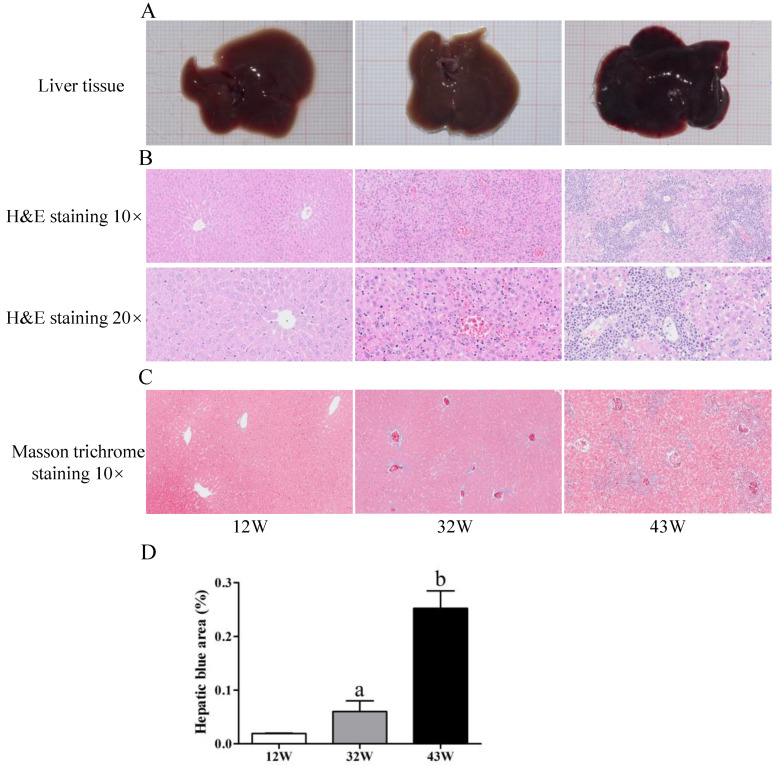
Aging induces liver fibrosis in mice. (**A**) Observation of the liver from the experimental mice. (**B**) Pathology observation of liver sections stained with H&E staining (10× and 20×). (**C**) Detection of collagen fibers by Masson’s trichrome staining (10×). (**D**) Quantitation of collagen fibers in liver (100%). The results are presented as mean ± SEM, and the different letters (a,b) represent significant differences compared to 12 W (*p* < 0.05).

Upon morphological examination, noticeable liver tissue damage was observed in mice at 32 W and 43 W (Figure 1A). Subsequently, an assessment of liver fibrosis was conducted, involving H&E and Masson’s trichrome staining. As shown in Figure 1B, the H&E staining revealed that the hepatocytes of 12 W mice were indicative of the S0 stage of fibrosis, portraying an intact lobular structure with normal morphology, clear central veins, radial distribution of hepatic cords, and no signs of necrosis or fibrosis surrounding the central veins. In the 32 W mouse group, the liver exhibited slight swelling, a feature not observed in 12 W mice. The architecture of the hepatic lobule was intact, the structure of chloasma hepaticum appeared unclear, and the structure of the portal vein was still discernible. Additionally, the hepatocytes displayed vacuolar degeneration, although fibrous septa were not evident. The pathological stage of the 32 W mice was denoted as S1. In comparison to the liver tissues from 12 W and 32 W mice, the hepatocytes of 43 W mice exhibited vacuolar degeneration, and the extensive formation of fibrous septa disrupted the architecture, although hepatic cirrhosis was not detected (S3 stage). In addition, collagen fibers were found to be scant in the livers of 12 W mice and most abundant in the livers of 43 W mice (*p* < 0.05) (Figure 1C,D).

### 3.2. Aging Increased EMT and Accumulation of Collagen in Mouse Liver

**Figure 2:**
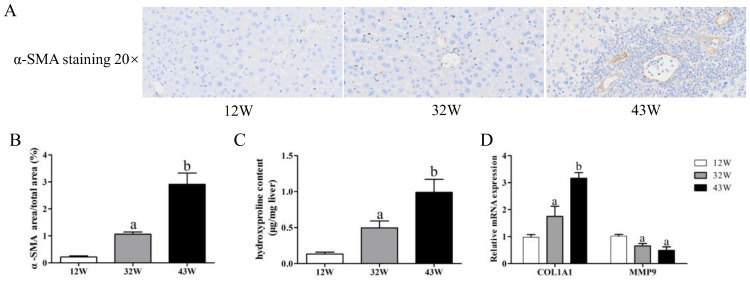
Effects of aging on EMT and accumulation of collagen in the liver. (**A**,**B**) Immunohistochemical analysis of α-SMA expression level (20×). (**C**) Hydroxyproline content in liver tissue. (**D**) Changes in relative expression of COL1A1 and MMP9 genes. The results are presented as mean ± SEM, and the different letters (a,b) represent significant differences compared to 12 W (*p* < 0.05).

It is known that the development of liver fibrosis is accompanied by EMT and an accumulation of collagen. Hence, our initial focus was on detecting changes in the expression of the myofibroblast marker α-SMA, which serves to indicate EMT. IHC from 12 W mice showed scarce α-SMA staining, whereas the liver tissue from 32 W mice was moderately stained, and that of 43 W mice was extensively stained (Figure 2A,B). On the other hand, we assessed the hydroxyproline content in liver tissues, observing a significant elevation in 32 W mice compared to 12 W mice (*p* < 0.05), and this elevation persisted in 43 W mice (*p* < 0.05) (Figure 2C). Quantitative analysis demonstrated that aging led to a remarkable increase in COL1A1 mRNA levels (*p* < 0.05), resulting in a reduced MMP9 mRNA level (*p* < 0.05) (Figure 2D).

### 3.3. Accelerated Cellular Senescence in Mouse Liver

**Figure 3:**
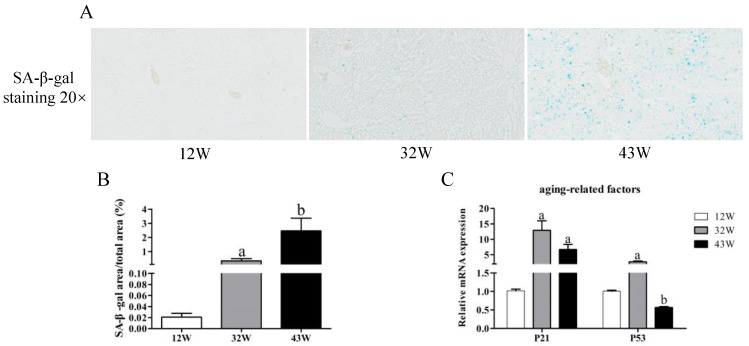
Detection of cellular senescence. (**A**,**B**) SA-β-gal staining (turquoise blue) of liver sections from mice of different ages was quantified (20×). (**C**) Changes in relative expression of aging-related genes P21 and P53. The results are presented as mean ± SEM, and the different letters (a,b) represent significant differences compared to 12 W (*p* < 0.05).

To evaluate cellular senescence, we quantified the intensity of SA-β-gal staining across mice of varying ages. The findings demonstrated a remarkable increase in SA-β-gal intensity within the livers of 32 W and 43 W mice compared to 12 W mice (*p* < 0.05), with the highest intensity observed in the 43 W mice, signifying an age-associated elevation in SA-β-gal activity (Figure 3A,B). Moreover, qPCR analysis showed that aging led to a significant increase in expression of the P21 gene (*p* < 0.05) (Figure 3C). Hepatic mRNA expression of P53 displayed marked augmentation in the 32 W mice, whereas it was suppressed in the 43 W mice, in contrast to the 12 W mice (*p* < 0.05).

### 3.4. Mitochondrial Dysfunction and Decreased Antioxidant Capacity in Mouse Liver

**Figure 4:**
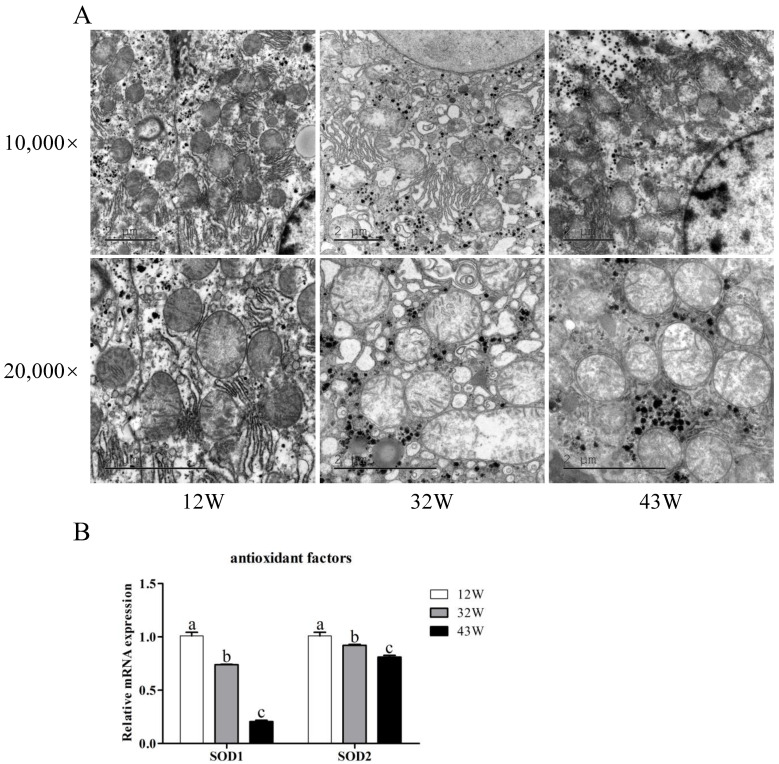
Effects of aging on mitochondrial function and antioxidant capacity in the liver. (**A**) Ultrastructure assessed by transmission electron microscopy (10,000× and 20,000×) of the liver tissue. (**B**) Changes in relative expression of antioxidant-related genes SOD1 and SOD2. The results are presented as mean ± SEM, and the different letters (a,b,c) represent significant differences compared to 12 W (*p* < 0.05).

Given that liver fibrosis compromises mitochondrial function and impacts the antioxidant capacity of hepatocytes, we proceeded to investigate mitochondrial morphology by using TEM and the expression of genes related to antioxidant function. TEM results showed diverse hepatocyte mitochondrial morphologies. In 12 W mice, hepatocytes exhibited normal morphology, with clearly visible and regularly arranged mitochondrial cristae (Figure 4A). Conversely, the 32 W group displayed an abundance of lipid droplets within hepatocytes, accompanied by unevenly sized and deformed mitochondria, as well as partial loss of mitochondrial cristae. These alterations were notably exacerbated in 43 W mice, where cytoplasmic vacuolization, substantial deterioration of mitochondrial morphology, and the disappearance of mitochondrial cristae were evident. Among the genes related to antioxidant function, the expression of SOD1 and SOD2 significantly declined with age, highlighting that aging impairs the antioxidant capacity of the liver (Figure 4B).

### 3.5. Expression Changes in Genes Related to Liver Fibrosis in Mouse Liver

**Figure 5:**
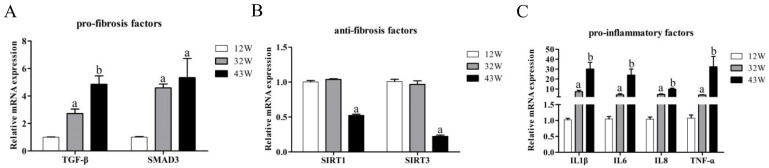
Aging modulates the expression of genes related to fibrosis in the liver of mice of different ages. (**A**) Effects of aging on the expression levels of genes related to the pro-fibrosis factors. (**B**) Effects of aging on the expression levels of genes related to the anti-fibrosis factors. (**C**) Effects of aging on the expression levels of genes related to the pro-inflammation factors. The results are presented as mean ± SEM, and the different letters (a,b) represent significant differences compared to 12 W (*p* < 0.05).

Finally, we explored the impact of aging on the expression of genes encoding other factors associated with liver fibrosis. Among the pro-fibrosis-related coding genes, the expression of both TGF-β and SMAD3 genes displayed an age-associated increase (*p* < 0.05) (Figure 5A). It is worth noting that although the expression of the SMAD3 gene in 43 W mice was higher than that in 32 W mice, it did not achieve statistical significance (*p* > 0.05). On the other hand, the expression of SIRT1 and SIRT3 genes was not significantly altered between 12 W and 32 W mice (*p* > 0.05); however, their expression was dramatically suppressed in 43 W mice (*p* < 0.05) (Figure 5B). As for the pro-inflammatory factor-coding genes IL1β, IL6, IL8, and TNF-α, aging notably enhanced their expression, peaking in the livers of 43 W mice (*p* < 0.05) (Figure 5C).

## 4. Discussion

The significant advancements in social economy and medical advancements over the past century have led to a consistent increase in human life expectancy [6]. However, this prolonged life expectancy has been accompanied by a higher occurrence of age-related conditions like liver fibrosis. Although prior research has established that aging contributes to the progression of hepatitis C fibrosis and reduces liver efficacy in alcoholic hepatitis [16,17], the precise biological mechanisms behind these phenomena remain inadequately understood. The traditional modeling methods of liver fibrosis, such as carbon tetrachloride (CCl_4_) treatment, fail to completely reproduce the pathogenesis of liver fibrosis in the elderly. This study uniquely addresses this gap by establishing and evaluating a spontaneous liver fibrosis model in NOD/SCID mice induced by natural aging. Our study’s histological and pathophysiological findings closely mirror the liver fibrosis model induced by CCl_4_ in SD rats and cynomolgus monkeys [8,18], as well as the morphological characteristics and pathophysiology observed in human liver fibrosis, including liver regeneration following hepatocyte necrosis. Therefore, in terms of liver morphology, this study has successfully established a model of liver fibrosis caused by natural aging, yet additional indicators must be scrutinized to comprehensively assess this model’s validity.

Liver fibrosis constitutes a pathological progression marked by a fibrotic response, concomitant with EMT processes, wherein myofibroblasts are accountable for α-SMA expression and collagen deposition, ultimately triggering substantial modifications in liver architecture [19]. Consequently, α-SMA has gained recognition as an EMT marker [20]. Hydroxyproline, an amino acid primarily present in collagens, serves as the “gold standard” for assessing collagen accumulation in fibrotic tissues [12]. In the current study, the pathological process of liver fibrosis induced by aging was associated with EMT, in which increased expression of α-SMA was observed, suggesting that aging activates the EMT process. Furthermore, aging caused an accumulation of collagen, a hallmark of liver fibrosis, substantiated by intensified Masson staining and increased hydroxyproline levels. This was further confirmed by augmented expression of collagen type I alpha 1 (COL1A1) mRNA, which is consistent with the characterization of liver fibrosis in transgenic mice [21]. Matrix metalloproteinase 9 (MMP9), an endopeptidase responsible for ECM component disruption and protein degradation, exhibited reduced expression in a previous study on Thioacetamide injection-induced liver fibrosis in rats [22]. Analogously, MMP9 expression was attenuated in the later stages of the liver fibrosis model presented in this study, confirming hepatocyte impairment in ECM component degradation following aging.

Cellular senescence entails a permanent cessation of cell proliferation in response to various pressures, contributing to the onset of aging-related diseases [23]. Aging hepatocytes can propel liver fibrosis development. The β-galactosidase activity of senescent cells is enhanced at pH 6.0 and can be detected by SA-β-gal staining. The tumor suppressor gene P53 orchestrates oxidative stress and DNA damage responses, alongside prompting P21 activation, ultimately imposing cell cycle arrest and triggering cellular senescence [24]. In addition, sirtuin 1 (SIRT1) and SIRT3 are members of the mammalian sirtuin family. SIRT1 influences oxidative reactions and cell cycle regulation via target protein activity modulation primarily within the nucleus, whereas SIRT3 is closely intertwined with mitochondrial oxidative stress [25]. They are both anti-fibrotic proteins that can ameliorate liver fibrosis through different mechanisms [25,26]. In this study, hepatocytes in 32 W and 43 W mice evidenced significant senescence features, marked by augmented SA-β-gal staining, elevated P21 expression, and decreased SIRT1 and SIRT3 expression. Intriguingly, the unexpectedly subdued P53 expression in the liver tissue of 43 W mice merits attention. A conjecture is that this might relate to the presence of a substantial number of activated myofibroblasts in the liver tissue of these mice. Whether this is the case requires further experimental verification.

Hepatic stellate cells (HSCs), the predominant stromal cell type in the liver, undergo a transition to myofibroblasts during liver fibrosis [19]. Mitochondria are a major source of intracellular reactive oxygen species (ROS), and their dysfunction leads to the accumulation of ROS in cells, which is the fundamental mechanism of tissue damage related to aging [27]. ROS-induced oxidative stress in hepatocytes activates HSCs via paracrine and autocrine pathways, culminating in liver fibrosis [28]. Recognizing this, bolstering cellular antioxidant capacity holds immense potential in ameliorating liver fibrosis. Through electron microscopy, this study has highlighted the pronounced mitochondrial abnormalities in 43 W mice. Simultaneously, the antioxidant prowess of hepatocytes in these mice experienced compromise due to a marked reduction in SOD1 and SOD2 expression, aligning with outcomes observed in CCl_4_-induced liver fibrosis in rats [29].

The transforming growth factor β (TGF-β)-SMAD signaling pathway stands as a pivotal route governing EMC production and liver fibrosis, which not only activates HSCs but also induces apoptosis in hepatocytes [30]. A previous study has confirmed that TGF-β transgenic mice can be instrumental in establishing liver fibrosis models [21]. Within our established model of naturally aging 43 W mice, the expression of TGF-β saw significant elevation, mirrored by an upsurge in the downstream gene SMAD3′s expression—an alignment with elevated SMAD3 expression observed in IGFBPrP1-induced liver fibrosis [30]. Concurrently, aging augments susceptibility to hepatic inflammation, as validated by the anomalous elevation of genes encoding pro-inflammatory factors with aging in our study. Cumulatively, these findings underscore the successful establishment of a spontaneous liver fibrosis model in 43 W mice.

Despite the aforementioned advancements, it is important to acknowledge the limitations of our study. In this study, we only detected the changes in gene expression by qPCR but did not analyze the changes of protein expression. Since it is the protein that functions, there is potential for further improvement by verifying the level of protein expression in future studies. In addition, the mice in this study were all male, and the liver fibrosis of aged female mice also needs to be further explored.

## 5. Conclusions

This study effectively accomplished the establishment of a spontaneous liver fibrosis model induced by natural aging within the NOD/SCID mouse. Through comprehensive evaluation utilizing state-of-the-art fibrosis-related readouts, this model demonstrates striking resemblance to liver fibrosis models constructed by other methods, paralleling the traits observed in human liver fibrosis. The significance of this study is twofold: not only did it successfully establish a mouse model of liver fibrosis, but it also unveiled the initial insights into the mechanisms driving aging-induced liver fibrosis. The culmination of this research offers a sturdy foundation with potential implications for the treatment of liver fibrosis among the elderly population.

## Data Availability

The data analyzed during the current study are available from the corresponding author on reasonable request.

## Supplementary Materials

The following supporting information can be downloaded at: https://www.mdpi.com/article/10.3390/biology12121493/s1, Table S1: The primer of qPCR.
