# Supplementary material for: Establishment of a Spontaneous Liver Fibrosis Model in NOD/SCID Mice Induced by Natural Aging
Source: Biology (Basel). 2023 Dec 6;12(12):1493. doi: 10.3390/biology12121493 (PMC10740877; doi:10.3390/biology12121493)
Supplement: Supplementary file 1 [file biology-12-01493-s001.zip › Supplementary Table S1.pdf]

Table S1 The primer of qPCR

Genes	Primer sequences (5′-3′)
P21	F: CCTTGTCGCTGTCTTGCACTCTG<br>R: GCTGGTCTGCCTCCGTTTTCG
P53	F: CACAGCACATGACGGAGGTC<br>R: TCCTTCCACCCGGATAAGATG
SIRT1	F: GACCTCCCAGACCCTCAAGCC<br>R: GTGACACAGAGACGGCTGGAAC
SIRT3	F: GGCTCTATACACAGAACATCGAC<br>R: TAGCTGTTACAAAGGTCCCGT
IL1β	F: TGGCAACTGTTCCTGAACTCAACTG<br>R: TCATCTTTTGGGGTCCGTCAACTTC
IL6	F: TAGTCCTTCCTACCCCAATTTCC<br>R: TTGGTCCTTAGCCACTCCTTC
IL8	F: ACCTGCTCTGTCACCGATGTCTAC<br>R: CAGGCAAGGTCAGGGCAAAGAAC
TNF-α	F: CAGGCGGTGCCTATGTCTC<br>R: CGATCACCCCGAAGTTCAGTAG
TGF-β	F: ACAATGGCGGTGCGGTCAAG<br>R: CAGACTTCATGCGGCTTCTCACAG
SMAD3	F: CACGCCTCACAGCCATCCATG<br>R: TCCCAATGTGTCGCCTTGTAAGTTC
SOD1	F: AGAGCATTCCATCATTGGCCGTAC<br>R: CGCAATCCCAATCACTCCACAGG
SOD2	F: CAATCTCAACGCCACCGAGGAG<br>R: AGGGCTCAGGTTTGTCCAGAAAATG
ACTB	F: GTGACGTTGACATCCGTAAAGA<br>R: GCCGGACTCATCGTACTCC
